# Introduction to virtual special issue on glucagon-like peptide-1 agonists and the skin

**DOI:** 10.1016/j.jdcr.2026.07.005

**Published:** 2026-07-08

**Authors:** Nikki A. Levin

**Affiliations:** Department of Dermatology, University of Massachusetts Chan School of Medicine, Worcester, Massachusetts

**Keywords:** case reports, GLP-1 receptor agonists

To the Editor:

Glucagon-like peptide-1 receptor agonists (GLP-1 RAs) such as liraglutide, semaglutide, and dulaglutide and the dual glucose-dependent insulinotropic polypeptide and glucagon-like peptide-1 (GLP-1) receptor co-agonist tirzepatide have become mainstay drugs for treating obesity and type 2 diabetes mellitus. In recent years, their use has spread far beyond the treatment of cardiometabolic disease to encompass virtually all areas of medicine, including potential uses in pulmonology, hepatology, rheumatology, nephrology, psychiatry/addiction medicine, and many other fields.

Dermatology is no exception. Evidence from clinical trials, cohort studies, and case reports suggests that GLP-1 RAs may positively impact psoriasis, hidradenitis suppurativa, wound healing, skin inflammation, and skin aging.^1^, ^2^, ^3^ In some cases, dermatologic improvement seems to track with weight loss, but in other cases, changes to the cytokine profile may improve skin disease even without weight loss.

In this issue, we present case reports suggesting that GLP-1 RAs may play a role in other challenging skin conditions, including Hailey-Hailey disease and erythromelalgia. Velusamy et al^4^ report a man with a 15-year history of treatment-refractory Hailey-Hailey disease whose eruption resolved within 2 months of starting tirzepatide, accompanied by an approximately 9-kg weight loss. Laugsand et al^5^ present the case of a woman with a 4-year history of erythromelalgia recalcitrant to aspirin and misoprostol whose symptoms resolved within 4 weeks of starting semaglutide for obesity, after losing only 2 kg.

Although GLP-1 RAs have revolutionized whole fields of medicine, they are not without side effects, the most common being gastrointestinal. Cutaneous side effects were also recognized early on, including injection-site reactions, hair loss, urticaria, dermatitis, and altered appearance due to loss of subcutaneous fat.^1^^,^^2^ In this issue, new cutaneous adverse effects reported range from hypersensitivity reactions to vascular, granulomatous, and subcutaneous fat disorders.

Some of the highlights include the case of a patient with diabetes on tirzepatide for 1 year who developed a diffuse pruritic eczematous eruption with biopsy showing eosinophil-rich spongiotic dermatitis.^6^ The eruption flared after tirzepatide injections and resolved after the drug was discontinued. Another patient developed a photodistributed eruption with biopsy showing spongiotic, psoriasiform, and interface dermatitis with eosinophils and purpura 2 months after starting semaglutide.^7^ The rash resolved after discontinuation. A patient on tirzepatide for weight loss developed an itchy rash in the axillae, antecubital fossae, inguinal folds, inframammary area, thighs, and periumbilical region, which was consistent with symmetrical drug-related intertriginous and flexural exanthema 3 weeks after starting the drug.^8^ The eruption resolved without treatment 1 month after discontinuation. Xu et al^9^ report a woman who developed a widespread pruritic eruption with facial swelling, lymphadenopathy, headaches, fatigue, intermittent chills, and fever 2 weeks after starting a GLP-1 mimetic supplement for weight loss. The biopsy showing spongiotic dermatitis with eosinophils, mildly elevated alanine aminotransferase (ALT), and blood eosinophilia suggested drug reaction with eosinophilia and systemic symptoms/Drug-Induced Hypersensitivity Syndrome. Discontinuation of the supplement and a prednisone taper led to resolution of the rash and normalization of her ALT and eosinophils.

Moore et al^10^ report a patient in whom persistent subcutaneous nodules developed at the sites of filler and neurotoxin injections while on compounded tirzepatide, though she had previously received the same injections without incident before starting the drug.^10^ Finally, Howard et al^11^ report a case of symptomatic papular granuloma annulare associated with tirzepatide that rapidly cleared upon discontinuation.

Of course, individual case reports can only show an association rather than a cause-and-effect relationship, but they suggest promising avenues for further research. As our experience with GLP-1 RAs grows, additional cases may accumulate that will point to which skin conditions these drugs may successfully treat and which cutaneous side effects are, in fact, drug related. We hope that you will enjoy this issue.

## Conflicts of interest

None disclosed.
